# Case Report: Pediatric mediastinal actinomycosis mimicking lymphoma diagnosed by tissue metagenomic next-generation sequencing

**DOI:** 10.3389/fped.2026.1882250

**Published:** 2026-07-06

**Authors:** Meng Yi, Ying Dai, Chao Liu, Huiping Lang, Xianping Jiang, Xiuli Yuan

**Affiliations:** 1 Department of Hematology and Oncology, Shenzhen Children’s Hospital, Shenzhen, Guangdong, China; 2 Department of Pathology, Shenzhen Children’s Hospital, Shenzhen, Guangdong, China

**Keywords:** case report, child, lymphoma mimic, mediastinal actinomycosis, metagenomic next-generation sequencing, necrotizing granuloma

## Abstract

Mediastinal actinomycosis is rare in children, and when it presents as a mass-like lesion, its clinical and imaging features overlap substantially with lymphoma, making differential diagnosis extremely challenging. We report a 2-year-1-month-old girl admitted with fever and cough. Contrast-enhanced chest computed tomography (CT) showed multiple enlarged mediastinal and bilateral hilar lymph nodes coalescing into a mass-like lesion with heterogeneous enhancement and small hypoenhancing foci, encasement of adjacent mediastinal vessels, and compression of the left main bronchus and the origin of the lingular bronchus. Magnetic resonance imaging (MRI) demonstrated heterogeneous signal intensity and enhancement; the radiologic differential included lymphoproliferative and granulomatous disease. Bone marrow biopsy, leukemia immunophenotyping, and tumor markers did not support malignancy. Ultrasound-guided biopsy of the mediastinal lesion revealed necrotizing granulomatous inflammation. Metagenomic next-generation sequencing (mNGS) of unstained tissue sections detected *Actinomyces oris* with mixed oropharyngeal flora, while *Mycobacterium tuberculosis* complex, fungi, viruses, and atypical pathogens were not detected. Pulmonary inflammation improved with antimicrobial therapy; however, repeat CT on January 28, 2026 showed little change in the mediastinal–hilar lesions. Because lymphoma could not be excluded, thoracoscopic partial resection was performed at another hospital, and postoperative pathology again showed granulomatous inflammation with caseous necrosis and negative acid-fast staining. Oral amoxicillin–clavulanate was continued postoperatively, in line with the principle of 2–6 weeks of intravenous therapy followed by 6–12 months of oral antibiotics for thoracic actinomycosis, with duration individualized to residual disease, imaging response, and drug tolerance. Follow-up ultrasonography on April 13, 2026 demonstrated reduction of the residual lesion, and the patient remained asymptomatic. This case highlights that pediatric mediastinal actinomycosis can mimic lymphoma and that integrated assessment of deep-tissue pathology, mNGS, serial imaging, and treatment response can guide diagnostic and therapeutic decision-making, preventing misdiagnosis and mistreatment.

## Introduction

Actinomycosis is a chronic suppurative granulomatous disease caused by *Actinomyces* species. These organisms are commensals of the oropharynx, gastrointestinal tract, and genitourinary tract, and are considered opportunistic pathogens. Disruption of the mucosal barrier, local tissue injury, aspiration, or mixed anaerobic co-infection can permit transition from colonization to invasive disease. Thoracic actinomycosis is uncommon overall, accounting for approximately 15%–20% of all actinomycosis cases; pediatric cases are rare, and primary or predominantly mediastinal disease is particularly unusual (8–10). Mediastinal actinomycosis can present with a mass-like lesion, fibrotic infiltration, necrotizing granulomatous inflammation, vascular encasement, and airway compression. Its clinical and imaging features overlap substantially with those of lymphoma, tuberculosis, invasive fungal disease, and other mediastinal tumors (1–4, 11).

Pediatric mediastinal masses pose a particular diagnostic challenge because both infectious and neoplastic etiologies can produce elevated inflammatory markers, airway compression, vascular involvement, and mass-like enhancement. Initial clinical and imaging findings alone are often insufficient to distinguish between them. In young children, a mediastinal lesion involving the airway or major vessels requires prompt exclusion of lymphoma (6). At the same time, granulomatous inflammation on tissue pathology or an oropharyngeal polymicrobial pattern on microbiological testing should prompt consideration of an infectious granulomatous disease, even when the lesion appears tumor-like. However, literature on pediatric mediastinal actinomycosis is extremely limited (16, 17), and clinicians’ awareness of this entity is inadequate, leading to a high risk of misdiagnosis as malignancy with consequent delayed treatment, or unnecessary aggressive surgery resulting in increased trauma to the child.

This case is unique because it demonstrates the diagnostic dilemma of pediatric mediastinal actinomycosis masquerading as lymphoma in a very young child, and illustrates how tissue mNGS can provide critical pathogen-level evidence when conventional microbiological methods are negative, thereby avoiding unnecessary radical surgery or chemotherapy. We describe a young child with mediastinal actinomycosis whose presentation initially mimicked lymphoma; the diagnosis was supported by deep-tissue pathology, mNGS, additional surgical sampling, and clinical follow-up. We focus on the differential diagnosis, interpretation of mNGS findings, timing of surgical intervention, and antimicrobial duration in this clinical scenario.

## Case description

A 2-year-1-month-old girl (weight, 12 kg; height, 87 cm) was admitted for a 9-day history of fever and cough. The peak temperature was 40 °C, recurring every 4–5 h, accompanied by chills and a transient lower-limb rash. She had a paroxysmal nonproductive cough but no wheezing, dyspnea, vomiting, diarrhea, abdominal pain, seizures, or weight loss. Her past medical history was otherwise unremarkable, with no recurrent or severe infections and no known tuberculosis exposure. Additional history confirmed no animal contact, plant or soil injury, unusual food exposure, dental surgery, oral trauma, or foreign-body aspiration; no obvious immunodeficiency was suspected clinically. Two weeks before the current presentation, she had been treated for bronchopneumonia at an outside hospital. Respiratory pathogen testing had been positive for *Streptococcus pneumoniae* DNA and influenza A virus RNA, and she improved with ceftriaxone and oseltamivir. During the current illness, she was readmitted to the outside hospital, where the leukocyte count and C-reactive protein were elevated and chest CT showed bronchopneumonia with a mediastinal soft-tissue shadow. Fever episodes became less frequent with antimicrobial and antiviral therapy but did not resolve, suggesting a possible deep-seated occult infectious focus or co-infection with additional pathogens.

On admission to our hospital, vital signs were as follows: temperature, 36.4 °C; heart rate, 127 beats/min; respiratory rate, 28 breaths/min; and oxygen saturation, 97% on room air. She was alert. The pharynx was congested, both tonsils were grade I enlarged, and no superficial lymphadenopathy was palpable. Breath sounds were coarse bilaterally without dry or moist rales. Cardiac, abdominal, and neurologic examinations were unremarkable; the extremities were warm, and capillary refill time was 2–3 s.

Laboratory studies showed serum amyloid A of 339.1 mg/L (markedly elevated) and mild elevations of interleukin-6, interleukin-10, and interferon-gamma, indicating active acute inflammation. Autoantibody profile, antinuclear antibody, and vasculitis-associated antibodies were negative. T-cell, B-cell, and NK-cell subsets were within age-appropriate ranges and did not suggest overt cellular immunodeficiency. Epstein–Barr virus DNA was <4.0 × 10^2^ copies/mL; EBV nuclear antigen IgG and viral capsid antigen IgG were positive while IgM was negative, consistent with prior infection and arguing against EBV-associated lymphoproliferative disease. Tumor markers (including alpha-fetoprotein, carcinoembryonic antigen, and neuron-specific enolase), thyroid function, and pituitary-related hormones were unremarkable. Tuberculosis-related immunologic screening (tuberculin skin test and interferon-gamma release assay) was negative, and the neutrophil respiratory burst test was normal, arguing against chronic granulomatous disease.

**Figure 1:**
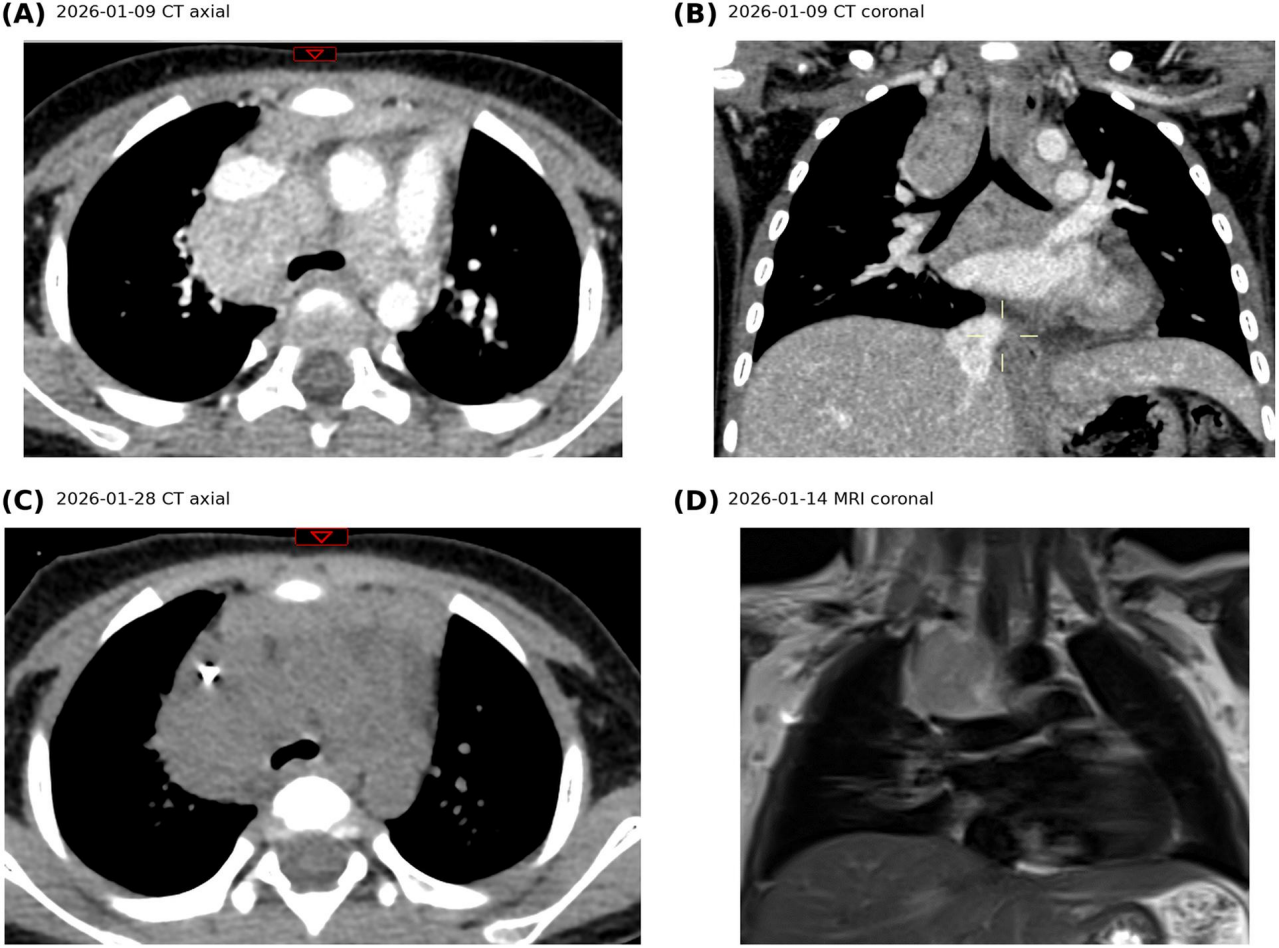
Representative imaging findings during hospitalization. **(A)** Axial contrast-enhanced chest CT on January 9, 2026 showing a mediastinal–hilar mass-like lesion with heterogeneous enhancement and airway compression. **(B)** Coronal contrast-enhanced CT on January 9, 2026 showing the relationship of the lesion to the airway, hila, and adjacent great vessels. **(C)** Axial non-contrast chest CT on January 28, 2026 showing persistent mediastinal–hilar disease without obvious reduction compared with January 9. **(D)** Coronal chest MRI on January 14, 2026 showing the mediastinal and hilar extent of disease. All images have been de-identified.

On January 9, 2026, contrast-enhanced chest CT showed multiple enlarged mediastinal and bilateral hilar lymph nodes coalescing into a mass-like lesion with heterogeneous enhancement and small hypoenhancing foci. The middle–posterior mediastinal and bilateral hilar lesion measured approximately 67 × 26 × 79 mm, encased adjacent mediastinal vessels, mildly narrowed the left main bronchus, and markedly compressed the origin of the lingular bronchus of the left upper lobe (Figures 1A,B). On January 14, 2026, chest MRI demonstrated an irregular mass-like lesion in the right middle–posterior mediastinum and bilateral hila with heterogeneous signal intensity and enhancement; the radiologic differential included granulomatous and lymphoproliferative disease (Figure 1D). Bilateral bone marrow biopsy and leukemia immunophenotyping showed no evidence of hematolymphoid malignancy, and flow cytometry revealed no abnormal lymphocyte populations. The overall clinical course and key decision points are summarized in Table 1.

**Table 1:** Main clinical timeline.

Date	Clinical event	Diagnostic or decision-making implication
2025-12-30	Onset of fever (peak 40 °C) and cough.	An acute respiratory infection was evident; however, the subsequent mediastinal–hilar mass-like lesion could not be explained by uncomplicated pneumonia alone.
2026-01-02 to 2026-01-07	Ceftriaxone and oseltamivir were administered at an outside hospital; fever episodes became less frequent but persisted.	Persistent inflammation suggested an uncontrolled or unrecognized focus.
2026-01-09 to 2026-01-14	Contrast-enhanced CT and MRI revealed mediastinal and bilateral hilar lesions with heterogeneous enhancement, small hypoenhancing foci, vascular encasement, and airway compression.	Lymphoma, infectious granulomatous disease, and other mediastinal tumors required consideration.
2026-01-10	Ultrasound-guided biopsy of the mediastinal lesion was performed.	Histology showed necrotizing granulomatous inflammation without definite malignancy.
2026-01-16 to 2026-01-17	Tissue mNGS was performed on unstained sections from the biopsy specimen.	Actinomyces oris with mixed oropharyngeal flora was detected; M. tuberculosis complex, fungi, viruses, and atypical pathogens were not detected.
2026-01-28 (≈4 weeks of antibiotics)	Repeat CT showed no obvious change in the mediastinal–hilar lesions despite improvement of pulmonary inflammation.	Slow radiologic regression did not exclude infection; however, persistent concern for malignancy required additional tissue confirmation.
2026-02-09	Thoracoscopic partial resection of the mediastinal lesion was performed at an outside hospital.	Histology again showed granulomatous inflammation with caseous necrosis; acid-fast staining was negative.
Postoperative period	Oral amoxicillin–clavulanate was continued as step-down therapy.	Treatment followed the long-course principle for thoracic actinomycosis (2–6 weeks of intravenous therapy followed by 6–12 months of oral antibiotics), with duration individualized to clinical and imaging response.
2026-04-13	Follow-up thoracic and mediastinal ultrasonography.	The residual right upper mediastinal lesion measured approximately 2.2 × 2.7 × 2.3 cm and had decreased in size; the patient remained asymptomatic.

## Diagnostic assessment, treatment, and follow-up

**Figure 2:**
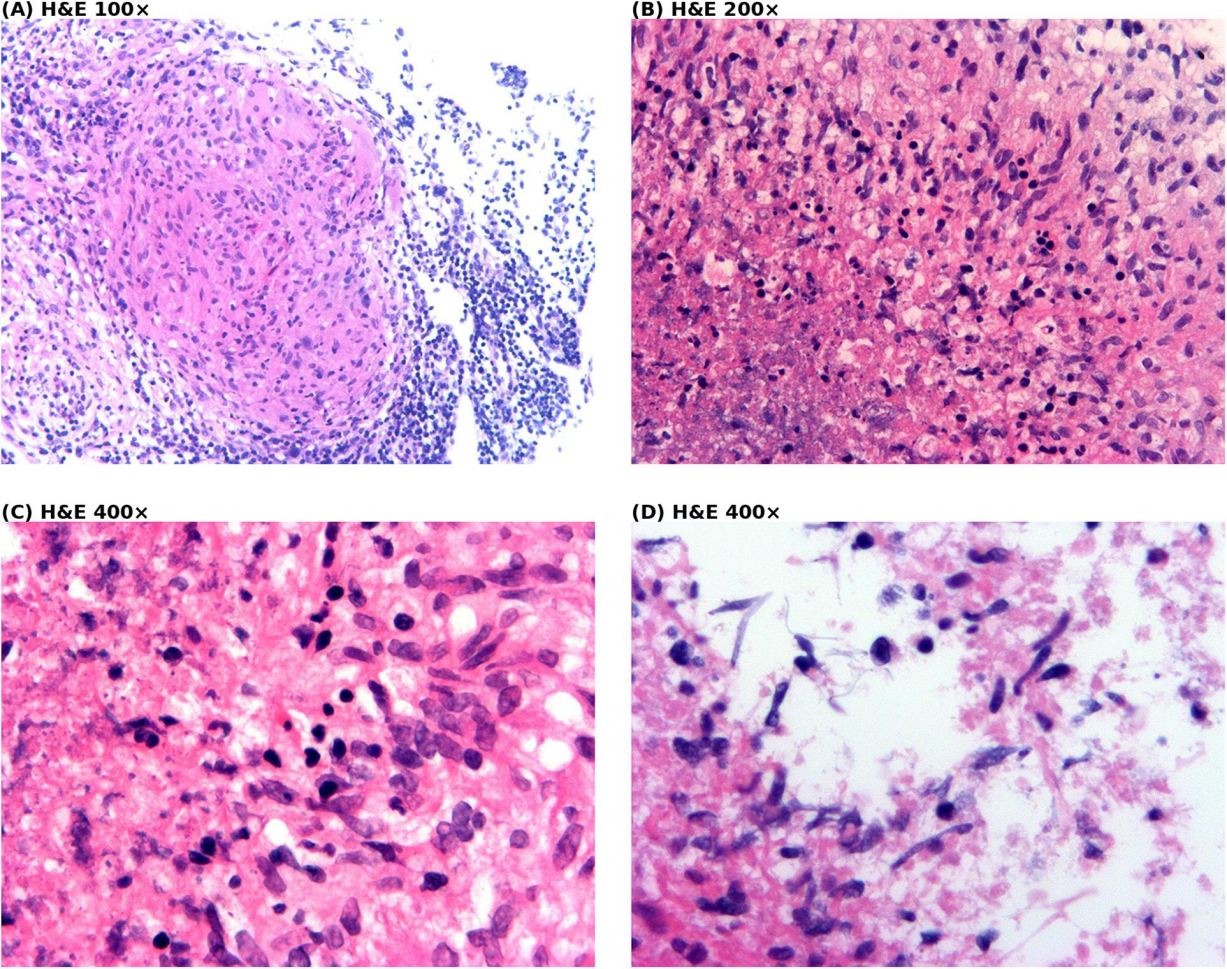
Pathology of the initial ultrasound-guided mediastinal biopsy. **(A)** Hematoxylin and eosin (H&E) staining, 100×. **(B)** H&E staining, 200×. **(C,D)** H&E staining, 400×. The tissue sections show necrotizing granulomatous inflammation without definite malignant cells. Postoperative pathology after thoracoscopic partial resection is described in the text.

After multidisciplinary discussion, ultrasound-guided biopsy of the mediastinal lesion was performed. Histology showed necrotizing granulomatous inflammation without definite malignant cells (Figure 2). mNGS of unstained tissue sections from the biopsy specimen detected multiple opportunistic bacteria of oropharyngeal origin. *Actinomyces* accounted for 43,563 reads at the genus level (relative abundance, 4.581%) and *Actinomyces oris* for 12,994 reads at the species level. *Streptococcus mitis*, *Haemophilus parainfluenzae*, *Neisseria elongata*, *Gemella haemolysans*, and *Granulicatella elegans* were also detected (Table 2). Fungi, DNA viruses, RNA viruses, parasites, *Mycobacterium tuberculosis* complex, *Mycoplasma*, *Chlamydia*, spirochetes, and rickettsiae were not detected. Internal and negative control results were acceptable, and the Q30 value was 95.343%.

**Table 2:** Major bacterial findings on mNGS of unstained biopsy tissue sections.

Genus/species	Gram stain	Genus-level reads	Genus-level relative abundance (%)	Species-level reads
*Streptococcus*/*Streptococcus mitis*	G+	430,142	45.235	46,570
*Haemophilus*/*Haemophilus parainfluenzae*	G−	164,083	17.255	33,945
*Neisseria*/*Neisseria elongata*	G−	66,867	7.032	20,696
*Gemella*/*Gemella haemolysans*	G+	53,247	5.600	48,678
*Actinomyces*/*Actinomyces oris*	G+	43,563	4.581	12,994
*Granulicatella*/*Granulicatella elegans*	G+	34,634	3.642	29,195

mNGS, metagenomic next-generation sequencing; G+, Gram-positive; G−, Gram-negative. Fungi, DNA viruses, RNA viruses, parasites, Mycobacterium tuberculosis complex, Mycoplasma, Chlamydia, spirochetes, and rickettsiae were not detected.

The patient had received ceftriaxone and oseltamivir at the outside hospital. Because fever and cough persisted and prior respiratory pathogen testing had been positive for *S. pneumoniae*, amoxicillin–clavulanate was initiated after admission. Once mNGS detected *A. oris* with mixed oropharyngeal flora, an Actinomyces-associated polymicrobial infection was considered, and treatment was continued with a beta-lactam/beta-lactamase inhibitor; duration was adjusted according to clinical course, imaging findings, and drug tolerance. Complete blood count, hepatic and renal function, and gastrointestinal adverse effects were monitored throughout therapy.

On January 17, 2026, repeat thoracic, mediastinal, and pulmonary ultrasonography demonstrated a mediastinal lesion of approximately 4.0 × 2.8 × 2.6 cm, indicating only minor reduction. On January 28, 2026, repeat non-contrast chest CT showed no obvious change in mediastinal and hilar lymphadenopathy compared with January 9 (Figure 1C). After approximately 4 weeks of antimicrobial therapy, pulmonary inflammation had improved, but regression of the mediastinal lesion remained unsatisfactory and intermittent fever persisted. Because the lesion remained mass-like, with vascular encasement and airway compression, and lymphoma could not be excluded, partial resection with biopsy was recommended. The family declined surgery at our hospital and requested discharge; the patient was transferred to another hospital and treated with piperacillin–tazobactam.

On February 9, 2026, thoracoscopic partial resection of the mediastinal lesion was performed at the outside hospital. The submitted specimen measured approximately 1 × 1 × 0.5 cm. Histology revealed coalescing nodular aggregates of epithelioid cells, some with central caseous necrosis, accompanied by multinucleated giant cells and lymphocytic infiltration; acid-fast staining was negative. After review with the pathologist, classical sulfur granules were not identified in the available tissue sections. Because caseous necrosis raised tuberculosis as an important differential diagnosis, the negative tuberculosis-related immunologic screening, negative acid-fast staining, absence of known tuberculosis exposure, and non-detection of Mycobacterium tuberculosis complex by tissue mNGS were considered together. The overall pathologic diagnosis was granulomatous inflammation with caseous necrosis, without evidence of malignancy. Oral amoxicillin–clavulanate was continued postoperatively as step-down therapy. Treatment followed the long-course principle for thoracic actinomycosis (2–6 weeks of intravenous therapy followed by 6–12 months of oral antibiotics), with subsequent duration individualized to symptoms, inflammatory markers, drug tolerance, and imaging resolution, given the large initial lesion, prior airway compression, partial rather than complete resection, and residual disease.

On April 13, 2026, follow-up ultrasonography at our hospital demonstrated a residual right upper mediastinal lesion of approximately 2.2 × 2.7 × 2.3 cm, smaller than on prior imaging. The patient had no fever, cough, chest tightness, or dyspnea, and her general status, appetite, and activity level were good. Oral amoxicillin–clavulanate was continued with regular follow-up. Because the follow-up was performed by ultrasonography rather than CT or MRI, percentage reduction relative to earlier cross-sectional imaging cannot be directly calculated; nevertheless, the reduction in lesion size and the absence of symptoms supported a positive treatment response.

## Discussion

This case illustrates a recurrent dilemma in pediatric mediastinal disease: clinical evidence of infection was present, yet the persistent mediastinal–hilar mass carried features that demanded exclusion of lymphoma. Fever, cough, and positive *S. pneumoniae* testing supported a respiratory infection. However, contrast-enhanced CT showed coalescing enlarged mediastinal and bilateral hilar lymph nodes with heterogeneous enhancement, small hypoenhancing foci, encasement of major vessels, and airway compression, and repeat CT on January 28 showed no obvious decrease. Uncomplicated bronchopneumonia could not account for this persistent mediastinal–hilar mass. In children, mediastinal lesions involving the airway or major vessels mandate prompt exclusion of lymphoma, with histologic confirmation when necessary.

Subsequent findings progressively weakened the lymphoma hypothesis. The patient had no superficial lymphadenopathy, tumor markers were unremarkable, and neither bone marrow cytology nor leukemia immunophenotyping supported hematolymphoid malignancy. MRI demonstrated a heterogeneous mass-like lesion, and the radiologic differential included both granulomatous and lymphoproliferative disease. The first biopsy showed necrotizing granulomatous inflammation. More extensive postoperative tissue sampling again demonstrated epithelioid cell nodules, caseous necrosis, multinucleated giant cells, and lymphocytic infiltration, without definite malignant cells; acid-fast staining was negative. Neither tissue sample was compatible with lymphoma, and the cumulative evidence against malignancy became substantial. However, caseous necrosis is more typical of tuberculosis, and although negative acid-fast staining argued against tuberculosis, it did not completely exclude nontuberculous mycobacterial infection or other granulomatous diseases. The negative mNGS results for *M. tuberculosis* complex played a critical exclusionary role in this regard.

Actinomycosis can mimic mediastinal lymphoma owing to its underlying pathobiology (7, 12). *Actinomyces* species commonly colonize the oropharynx and can form biofilms, synergizing with other oral flora to promote pathogenicity. Human actinomycosis usually arises from endogenous mucosal flora after mucosal barrier disruption, aspiration, local tissue injury, or mixed anaerobic infection, rather than from a typical food-, plant-, or animal-borne reservoir. In this patient, additional history confirmed no animal contact, plant/soil injury, unusual food exposure, dental surgery, oral trauma, or foreign-body aspiration. A precise portal of entry could not be established, but the deep-tissue mNGS pattern suggested an oropharyngeal endogenous source. A local low-oxygen environment or mixed bacterial infection can enable invasion into deep tissue, where chronic suppurative granulomatous inflammation, necrosis, microabscesses, and fibrosis develop. On imaging, the resulting lesions can appear as soft-tissue masses with heterogeneous enhancement, focal hypoenhancement or necrosis, and contiguous spread across adjacent tissue planes, leading to vascular encasement and airway compression (19). When the mediastinum is involved, these features overlap substantially with lymphoma, tuberculosis, and invasive fungal disease, and no single imaging study is sufficient for etiologic diagnosis. Pediatric cases are particularly difficult because normal thymic tissue in infants and young children can appear as anterior mediastinal soft-tissue shadows, further complicating the differential diagnosis of mediastinal masses.

The mNGS results were central to the differential diagnosis but cannot be reduced to a simple equation between detection of *A. oris* and a diagnosis of actinomycosis. *A. oris* is a component of the normal oropharyngeal flora, and low-level detection in sputum, throat swabs, or bronchial secretions may reflect colonization or contamination. In this patient, however, the specimen was an unstained tissue section from the mediastinal lesion, representing deep tissue rather than upper-airway material. *Actinomyces* accounted for 43,563 reads at the genus level and *A. oris* for 12,994 reads at the species level, which does not represent a low-level or incidental finding. The same specimen also yielded *S. mitis*, *H. parainfluenzae*, *N. elongata*, *G. haemolysans*, and *G. elegans—an oropharyngeal polymicrobial pattern consistent with the well-recognized polymicrobial nature of actinomycotic infection and compatible with this patient's prior history of oropharyngeal-respiratory infection. Fungi, viruses, *M. tuberculosis* complex, and atypical pathogens were not detected, and sequencing quality control was acceptable. Interpretation of such findings should involve multidisciplinary assessment, including microbiology and infectious disease expertise when available (13), because pathogen plausibility depends on specimen source, read abundance, background flora, negative controls, and clinicopathologic concordance. Together with necrotizing granulomatous inflammation on histology, a negative malignancy workup, clinical improvement on beta-lactam therapy, and reduction of the residual lesion at follow-up, the mNGS data are best interpreted as lesion-level pathogen evidence rather than as an isolated contamination signal. An important caveat is that neither anaerobic culture positivity nor classical sulfur granules were obtained; the mNGS result should therefore be interpreted within the full evidence chain that includes histopathology, imaging, exclusionary testing, and treatment response. Future studies could explore *Actinomyces*-specific primer PCR or 16S rRNA sequencing as supplementary validation methods.

The unsatisfactory regression of the mediastinal lesion after approximately 4 weeks of antimicrobial therapy was a critical decision point. In actinomycosis, radiologic resolution often lags behind clinical and laboratory improvement because the underlying lesion is composed largely of necrotic, granulomatous, and fibrotic tissue; persistence of a mass-like appearance on early follow-up imaging therefore does not, by itself, exclude an infectious etiology. Literature reports indicate that complete radiologic resolution of thoracic actinomycosis may require months or even longer; premature termination of therapy or misjudgment of treatment failure may lead to unnecessary surgery. Conversely, in this patient the lesion still encased major vessels and compressed the airway, intermittent fever persisted, and lymphoma had not been definitively excluded. Continued observation based only on the assumption of slow resolution could have delayed the diagnosis of malignancy or of another treatable disease. At that point, neither an infectious nor a neoplastic diagnosis could be accepted without further confirmation, and more representative tissue sampling was required to mitigate diagnostic risk. The multidisciplinary team played a pivotal role in timing this decision appropriately.

Thoracoscopic partial resection with biopsy was therefore appropriate. Surgery yielded substantially more tissue than the initial needle biopsy, reducing the risk that lymphoma, inflammatory myofibroblastic tumor, or another neoplasm would be missed because of sampling error. Partial resection additionally reduced lesion burden and the potential impact of persistent mass effect on the airway and great vessels, while alleviating parental anxiety. Postoperative pathology showed granulomatous inflammation with caseous necrosis and negative acid-fast staining, consistent with the earlier biopsy and mNGS findings and further weakening the case for malignancy. In this setting, surgery should not be viewed merely as salvage therapy after antimicrobial failure; rather, it served simultaneously as a diagnostic and therapeutic intervention when the initial biopsy could not fully account for the tumor-like imaging appearance, the lesion lay adjacent to critical structures, and the early radiologic response was unsatisfactory. For pediatric patients, thoracoscopic minimally invasive surgery is less traumatic and allows faster recovery than open thoracotomy, representing a reasonable choice that balances diagnostic needs with surgical safety.

The antimicrobial strategy paralleled the evolving diagnostic interpretation. Amoxicillin–clavulanate was started after admission because of fever, cough, pulmonary infection, and prior positive *S. pneumoniae* testing. Once mNGS identified *A. oris* with mixed oropharyngeal flora, continuation of a beta-lactam/beta-lactamase inhibitor was reasonable: amoxicillin covers *Actinomyces*, whereas clavulanate broadens coverage against beta-lactamase-producing organisms that frequently coexist in polymicrobial actinomycotic infection. Piperacillin–tazobactam, used before surgery at the outside hospital, was likewise consistent with coverage for a deep polymicrobial and anaerobic-associated infection, and additionally provided coverage against *Pseudomonas aeruginosa* and other nosocomial pathogens, which was rational in the outside hospital setting. Traditional treatment of thoracic actinomycosis consists of 2–6 weeks of intravenous antimicrobial therapy followed by 6–12 months of oral antibiotics, with duration individualized to lesion burden, completeness of surgical resection, clinical and imaging response, and drug tolerance (5, 14, 15, 18). Given the patient's young age, large initial mediastinal lesion, prior airway compression, partial rather than complete resection, and residual disease, 6–12 months of oral antibiotic therapy with regular follow-up was considered appropriate. Hepatic and renal function and gastrointestinal symptoms should be closely monitored during treatment, with dose adjustment or drug substitution as needed.

The limitations of this case include the absence of anaerobic culture positivity and classical sulfur granules, the lack of an independent validation method for the mNGS findings (such as 16S rRNA gene sequencing), and the use of ultrasonography rather than CT or MRI for follow-up, precluding precise calculation of percentage lesion reduction. The April 13, 2026 assessment represents an interim follow-up while antimicrobial treatment was still ongoing; therefore, complete cure, final antibiotic duration, and long-term radiologic outcome cannot yet be claimed. Furthermore, the thoracoscopic partial resection specimen was still relatively limited (1 × 1 × 0.5 cm), and the possibility of tumor components at the lesion margin cannot be completely excluded; continued follow-up until treatment completion and/or complete lesion resolution is needed.

**Figure 3:**
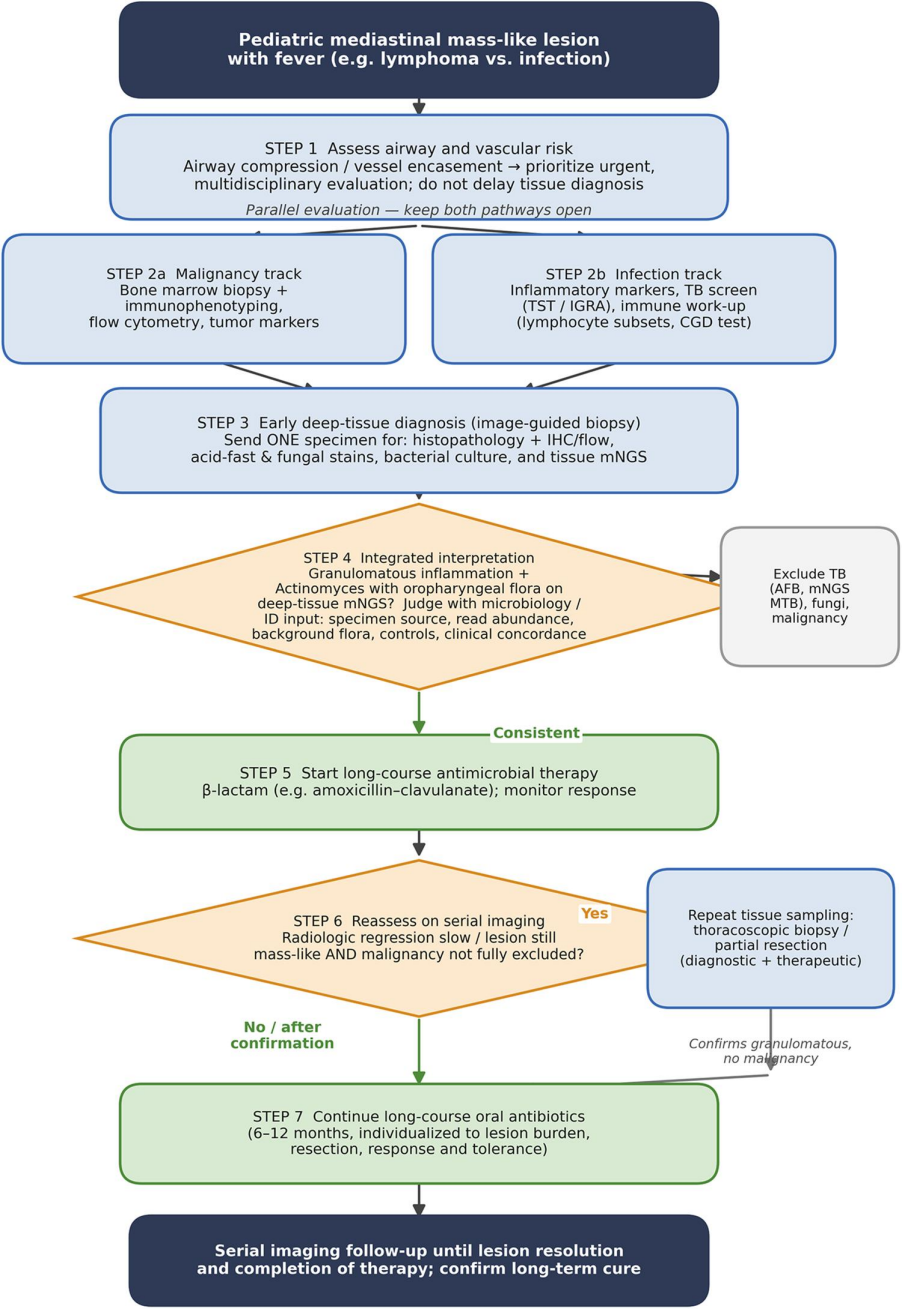
Diagnostic and therapeutic decision flow chart for pediatric mediastinal mass-like lesions when actinomycosis mimics lymphoma. The flow chart summarizes the stepwise approach suggested by this case: assessment of airway and vascular risk; parallel evaluation for malignancy and infection; early tissue diagnosis; integrated interpretation of granulomatous pathology and tissue mNGS with microbiology/infectious disease input; reassessment when radiologic regression is slow; consideration of repeat tissue sampling or thoracoscopic biopsy/partial resection when malignancy remains possible; and long-course antimicrobial treatment with serial imaging follow-up.

The principal clinical lesson is that fever accompanying a pediatric mediastinal mass should not force a premature choice between infection and malignancy; both diagnostic pathways should be kept open until sufficient tissue, microbiology, and clinical follow-up data are available. Early assessment should prioritize airway, vascular, and oncologic risk and should secure deep tissue as soon as feasible. Ideally, the same specimen should undergo histopathology, appropriate immunohistochemistry or flow cytometry, acid-fast and fungal studies, bacterial culture, and mNGS. When pathology shows granulomatous inflammation and mNGS detects *Actinomyces* with mixed oropharyngeal flora, pathogenic significance should be judged on specimen source, read count, accompanying flora, the panel of negative differential tests, and treatment response. Slow radiologic resolution after antimicrobial therapy should not automatically refute infection, but passive observation is unsafe when malignancy has not been excluded. When the lesion lies close to the airway or major vessels, when the initial biopsy does not fully account for the tumor-like appearance, or when lesion regression is unsatisfactory, timely thoracoscopic re-biopsy or partial resection should be considered. This approach can reduce two complementary errors: misclassifying infectious granulomatous disease as lymphoma, and delaying the diagnosis of true malignancy by treating it as infection. Future accumulation of additional pediatric mediastinal actinomycosis cases is needed to establish mNGS-based diagnostic criteria and individualized treatment protocols. A practical, step-by-step diagnostic and therapeutic decision pathway summarizing this approach is provided in Figure 3.

## Patient perspective

Because the patient was 2 years and 1 month old, she could not directly describe her illness experience. During diagnosis and treatment, the guardian's main concerns were whether the mediastinal lesion represented malignancy, whether long-term antimicrobial therapy was safe, and whether the residual lesion might recur. After the pathology, mNGS findings, and follow-up changes were explained, the guardian expressed understanding of the need to continue oral amoxicillin-clavulanate and regular follow-up.

## Data Availability

The original contributions presented in the study are included in the article. Because the case involves a minor, the raw metagenomic next-generation sequencing data are not publicly available in order to protect patient privacy; de-identified data may be made available by the corresponding author upon reasonable request, subject to ethical and privacy requirements. Further inquiries can be directed to the corresponding author.
